# A Predictive Rule for COVID-19 Pneumonia Among COVID-19 Patients: A Classification and Regression Tree (CART) Analysis Model

**DOI:** 10.7759/cureus.45199

**Published:** 2023-09-13

**Authors:** Sayato Fukui, Akihiro Inui, Takayuki Komatsu, Kanako Ogura, Yutaka Ozaki, Manabu Sugita, Mizue Saita, Daiki Kobayashi, Toshio Naito

**Affiliations:** 1 Department of General Medicine, Faculty of Medicine, Juntendo University, Tokyo, JPN; 2 Department of Emergency and Critical Care Medicine, Juntendo University Nerima Hospital, Tokyo, JPN; 3 Department of Diagnostic Pathology, Juntendo University Nerima Hospital, Tokyo, JPN; 4 Department of Diagnostic Radiology, Juntendo University Nerima Hospital, Tokyo, JPN; 5 Department of General Internal Medicine, Tokyo Medical University Ibaraki Medical Center, Inashiki, JPN

**Keywords:** severe acute respiratory syndrome coronavirus 2, clinical feature, computed tomography, blood testing, prevalent infection

## Abstract

Background: In this study, we aimed to identify predictive factors for coronavirus disease 2019 (COVID-19) patients with complicated pneumonia and determine which COVID-19 patients should undergo computed tomography (CT) using classification and regression tree (CART) analysis.

Methods: This retrospective cross-sectional survey was conducted at a university hospital. We recruited patients diagnosed with COVID-19 between January 1 and December 31, 2020. We extracted clinical information (e.g., vital signs, symptoms, laboratory results, and CT findings) from patient records. Factors potentially predicting COVID-19 pneumonia were analyzed using Student’s *t*-test, the chi-square test, and a CART analysis model.

Results: Among 221 patients (119 men (53.8%); mean age, 54.59±18.61 years), 160 (72.4%) had pneumonia. The CART analysis revealed that patients were at high risk of pneumonia if they had C-reactive protein (CRP) levels of >1.60 mg/dL (incidence of pneumonia: 95.7%); CRP levels of ≤1.60 mg/dL + age >35.5 years + lactate dehydrogenase (LDH)>225.5 IU/L (incidence of pneumonia: 95.5%); and CRP levels of ≤1.60 mg/dL + age >35.5 years + LDH≤225.5 IU/L + hemoglobin ≤14.65 g/dL (incidence of pneumonia: 69.6%). The area of the curve of the receiver operating characteristic of the model was 0.860 (95% CI: 0.804-0.915), indicating sufficient explanatory power.

Conclusions: The present results are useful for deciding whether to perform CT in COVID-19 patients. High-risk patients such as those mentioned above should undergo CT.

## Introduction

In December 2019, a series of pneumonia cases of unknown causes were reported, involving clinical presentations that greatly resembled viral pneumonia [1]. The coronavirus disease 2019 (COVID-19) pandemic has been a great threat to human life. The outbreak of coronavirus was initially reported to the World Health Organization on December 31, 2019 [2]. More than 45 million people have been infected with COVID-19, with 1.2 million deaths reported [2]. The most common method of diagnosing COVID-19 is using molecular genetic assays for the detection of viral RNA from a clinical sample using reverse transcription-polymerase chain reaction (RT-PCR) [3]. Many case reports on COVID-19 pneumonia have been published [4-6]. One study described chest computed tomography (CT) findings in 51 COVID-positive patients, with 77%, 75%, and 59% of cases showing pure ground-glass opacities (GGOs), GGOs with interstitial and/or interlobular septal thickening, and GGOs with consolidation, respectively [4]. Furthermore, the latest literature studies have led to the study of predicting the severity of hematologic and clinical findings from CT findings, which is an interesting topic [5,6].

Furthermore, COVID-19 symptoms that do not manifest in past viral infections (e.g., anosmia and ageusia) have also been reported [7,8]. The wide range of reported features is thought to reflect the effects of COVID-19 on non-respiratory systems and indicates that signs of the disease may be observed in infected patients without apparent respiratory symptoms [9]. Therefore, we must continuously manage COVID-19.

One study suggested that due to the COVID-19 epidemic/pandemic, focusing on the exclusion of its infection using CT scans leads to an overall delay in the diagnosis and treatment of bacteremia [10]. When COVID-19 pneumonia can be distinguished in conclusive cases from suspicious cases, unnecessary examinations (e.g., CT scans) can be avoided.

Unfortunately, predicting COVID-19 pneumonia is difficult compared to other forms of pneumonia. Although fever and decreased oxygenation are typical symptoms of pneumonia [11], as described above, asymptomatic cases may develop critical pneumonia [2]. Furthermore, the presence or absence of pneumonia is clinically important, even if asymptomatic, in affecting future follow-up policies, as pneumonia is assumed to aggravate this risk. However, the predictive factors of COVID-19 pneumonia, including clinical parameters with symptoms, vital signs, and laboratory data, have not been compared directly between cases with and without complicated pneumonia using decision tree analysis. Moreover, the presence of pneumonia on CT is also an important determinant in the decision to treat, and being able to predict whether pneumonia is present or not would be more meaningful in a clinical setting.

In this retrospective cross-sectional survey, we aimed to compare clinical parameters in patients with and without complicated pneumonia to identify predictive factors for COVID-19 patients with complicated pneumonia and determine which COVID-19 patients should undergo CT. Although the Centers for Disease Control and Prevention (CDC) has published the most recent findings regarding the underlying causes of COVID-19 severity at any time, we emphasize that our study is different and is a new predictive model.

## Materials and methods

Study design and population

In this retrospective cross-sectional survey performed at Juntendo University Nerima Hospital, a 490-bed university-affiliated hospital in Tokyo, Japan, we recruited patients diagnosed with COVID-19 between January 1 and December 31, 2020. The inclusion criteria were COVID-19 patients who underwent blood tests and CT scans. The exclusion criteria were refusal to participate in the study via opt-out notice by the institutional ethics committee and children aged <14 years with COVID-19. COVID-19 was diagnosed by more than two internists upon detection of severe acute respiratory syndrome coronavirus 2 (SARS-CoV-2) nucleic acids via RT-PCR.

We extracted clinical information through a chart review. We collected data on age, sex, history of malignant diseases, asthma, heart disease (including hypertension), diabetes mellitus, hemodyscrasia, human immunodeficiency virus infection, use of immunosuppressive agents (including steroids), and general symptoms (chills, malaise, joint pain, headache, nausea, diarrhea, smell disturbances, taste disturbances, sore throat, cough, and difficulty in breathing). We also extracted data on axillary body temperature, blood pressure, pulse rate, respiratory rate, oxygen saturation (room air), white blood cell count, percentage of neutrophils and lymphocytes, hemoglobin level, platelet count, red blood cell distribution width, serum parameters (total protein, albumin, lactate dehydrogenase (LDH), blood urea nitrogen, creatinine, sodium, potassium, chloride, glucose, aspartate aminotransferase (AST), alanine aminotransferase (ALT), total bilirubin, glucose, hemoglobin A1c, and C-reactive protein (CRP) levels), and CT findings of pneumonia.

Statistical analysis

We compared bi-variates in COVID-19 patients with and without complicated pneumonia using independent sample t-tests and chi-square tests for continuous and categorical data, respectively. To create a prediction model for COVID-19 patients with complicated pneumonia, we used the classification and regression tree (CART) methodology to identify patients at different levels of risk. The CART model is well suited to the generation of clinical decision rules and has been used to develop prediction models in various fields, including in medical settings [12]. The CART method employs a non-parametric statistical technique that makes no distribution assumptions of any kind, for either dependent or independent variables [13]. A decision tree is created by stratifying the initial dataset, which contains all potential predictors, into subsets based on the “impurity” of the model. The branch nodes in the decision are based on the “impurity” of the model [14], which is represented by the Gini diversity index (GI):

ΔGI(t) = PtGI(t) － PLGI(tL) － PRGI(tR),

where ΔGI(t) represents the variation of GI, GI(t) represents the Gini diversity index at node t, Pt represents the ratio of node t before partition, PL represents the ratio of the left node after partition, and PR represents the ratio of the right node after partition. The risk factor that maximizes impurity is selected as a branch point. This process is repeated for each derived subset until the impurity value of the subset is not improved by additional splitting [12].

In this study, the CART algorithm analysis included 48 potential variables. Nodes in the CART decision tree were constrained to a minimum size of 40 subjects to consider additional stratification, and each resulting subgroup required at least 20 subjects. A receiver operating characteristic (ROC) curve was drawn from the results of the CART analysis, and the area under the curve (AUC) was calculated to evaluate the accuracy of the tree. After arriving at the final decision tree, we performed cross-validation to derive the standard error of the branches of the CART model. All analyses were conducted using IBM SPSS Statistics for Windows, Version 27 (Released 2020; IBM Corp., Armonk, New York, United States)., except for the calculation of the 95% confidence intervals (CIs), which was based on an exact binominal using Stata version 16.1 (STATA Corp., College Station, USA) [15].

This study was conducted in accordance with the relevant guidelines and regulations and approved by the institutional ethics committee of Juntendo University, Tokyo, Japan (approval number: E21-0065-N05). This was an observational study, and written informed consent was waived in light of the public health outbreak investigation by the ethics committee.

## Results

Among 221 included patients in the study, 119 were men (53.8%), the mean age was 54.59 ± 18.61 years, and 160 (72.4%) had pneumonia. Table 1 shows the patient characteristics and the results of the bivariate analysis.

**Table 1:** Patient variables and results of bivariate analysis COVID-19, coronavirus disease 2019; SD, standard deviation

	Total	COVID-19 Pneumonia (+)	COVID-19 Pneumonia(-)		p-value
Variables	n=221	n=160	n=61	Name of test	
Demographic factors					
Age, year, ±SD	54.59±18.61	57.67±16.97	46.52±20.40	t-test	<0.001*
Female sex, n, %	102 (46.2%)	76 (47.5%)	26 (42.6%)	χ^2^	0.55
Underlying condition					
Cancer bearing, n, %	12 (5.4%)	10 (6.3%)	2 (3.3%)	χ^2^	0.52
Hemodyscrasia, n, %	6 (2.7%)	4 (2.5%)	2 (3.3%)	χ^2^	0.09
Diabetes mellitus, n, %	25(11.3%)	19 (11.9%)	6 (9.8%)	χ^2^	0.85
Asthma, n, %	28 (12.7%)	17 (10.6%)	11(18.0%)	χ^2^	0.21
Heart disease, n, %	57(25.8%)	48 (30.0%)	9 (14.8%)	χ^2^	0.03*
Human immunodeficiency syndrome, n, %	0	0	0	χ^2^	NA
Use of immunosuppressive agents, n, %	8 (3.6%)	5 (3.1%)	3 (4.9%)	χ^2^	0.81
Smoker, n, %	45 (20.4%)	31 (19.4%)	14 (23.0%)	χ^2^	0.79
Symptoms					
Headache, n, %	26 (11.8%)	19 (11.9%)	7 (11.5%)	χ^2^	1.00
Cough, n, %	94 (42.5%)	74 (46.3%)	20 (32.8%)	χ^2^	0.10
Sore throat, n, %	32 (14.5%)	17 (10.6%)	15 (24.6%)	χ^2^	0.02*
Difficulty in breathing, n, %	47 (21.3%)	40 (25.0%)	7 (11.5%)	χ^2^	0.04*
Chill, n, %	8 (3.6%)	7 (4.3%)	1 (1.6%)	χ^2^	0.57
Joint pain, n, %	16 (7.2%)	14 (8.8%)	2 (3.3%)	χ^2^	0.27
Nausea, n, %	3 (1.4%)	1(0.6%)	2 (3.3%)	χ^2^	0.38
Diarrhea, n, %	14 (6.3%)	11 (6.9%)	3 (4.9%)	χ^2^	0.82
Malaise, n, %	71 (32.1%)	48 (30%)	23 (37.7%)	χ^2^	0.35
Dysosmia, n, %	39 (17.6%)	27 (16.9%)	12 (19.7%)	χ^2^	0.77
Taste disturbance, n, %	37 (16.7%)	26 (16.3%)	11 (18.0%)	χ^2^	0.91
Vital signs					
Body temperature, ℃, ±SD	36.93±0.77	36.99±0.77	36.79±0.74	t-test	0.08
Systolic blood pressure, mmHg, ±SD	127.94±19.53	129.10±18.43	124.83±22.08	t-test	0.15
Diastolic blood pressure, mmHg, ±SD	79.41±14.49	80.16±14.34	77.37±14.83	t-test	0.21
Heart rate, bpm, ±SD	84.99±15.87	86.37±14.76	81.30±18.15	t-test	0.03*
Respiratory rate, n, ±SD	17.26±3.29	17.39±3.25	16.89±3.40	t-test	0.33
Saturation, %, ±SD	95.85±3.60	95.62±3.67	96.48±3.36	t-test	0.11
Laboratory data					
White blood cell counts, /μL, ±SD	5221.72±2252.41	5227.50±1949.74	5075.41±2915.17	t-test	0.55
Neutrophils, %, ±SD	63.94±13.89	65.89±13.32	58.93±14.20	t-test	<0.001*
Lymphocytes, %, ±SD	26.65±11.81	25.04±11.06	30.78±12.76	t-test	<0.01*
Hemoglobin, g/dL, ±SD	13.98±1.94	13.93±1.89	14.11±2.06	t-test	0.53
Red blood cell distribution width, %, ±SD	12.80±1.44	12.76±1.35	12.93±1.65	t-test	0.43
Platelet, 10^4^/μL, ±SD	21.66±9.07	21.58±9.61	21.87±7.53	t-test	0.83
Blood urea nitrogen, mg/dL, ±SD	15.19±10.62	15.21±10.13	15.15±11.90	t-test	0.97
Creatinine, mg/dL, ±SD	0.91±0.68	0.88±0.52	0.97±0.98	t-test	0.38
Total protein, g/dL, ±SD	7.08±0.68	7.05±0.66	7.16±0.76	t-test	0.32
Albumin, g/dL, ±SD	3.98±0.61	3.88±0.57	4.23±0.63	t-test	<0.001*
Total bilirubin, g/dL, ±SD	0.57±0.53	0.56±0.28	0.59±0.90	t-test	0.70
Lactate dehydrogenase, IU/L, ±SD	244.80±101.02	263.38±98.13	196.36±92.69	t-test	<0.001*
Aspartate aminotransferase, IU/L, ±SD	32.33±22.80	34.60±24.54	26.38±16.13	t-test	0.02*
Alanine aminotransferase, IU/L, ±SD	32.94±38.61	35.18±43.59	27.05±19.51	t-test	0.16
Sodium, mEq/L, ±SD	138.45±3.50	138.02±3.82	139.57±2.12	t-test	<0.01*
Potassium, mEq/L, ±SD	4.07±0.41	4.07±0.45	4.08±0.30	t-test	0.83
Chloride, mEq/L, ±SD	103.43±3.84	102.96±4.11	104.66±2.67	t-test	<0.01*
C-reactive protein, mg/dL, ±SD	3.12±4.67	3.86±4.67	1.18±4.11	t-test	<0.001*
Creatine Phosphokinase, U/L, ±SD	122.12±200.44	135.85±232.51	86.22±48.36	t-test	0.10
Glucose, mg/dL, ±SD	114.12±36.96	117.71±38.78	104.53±29.81	t-test	0.02*
Hemoglobin A1c, %, ±SD	6.04±0.88	6.10±0.90	5.90±0.81	t-test	0.17
*p＜0.05					

**Figure 1:**
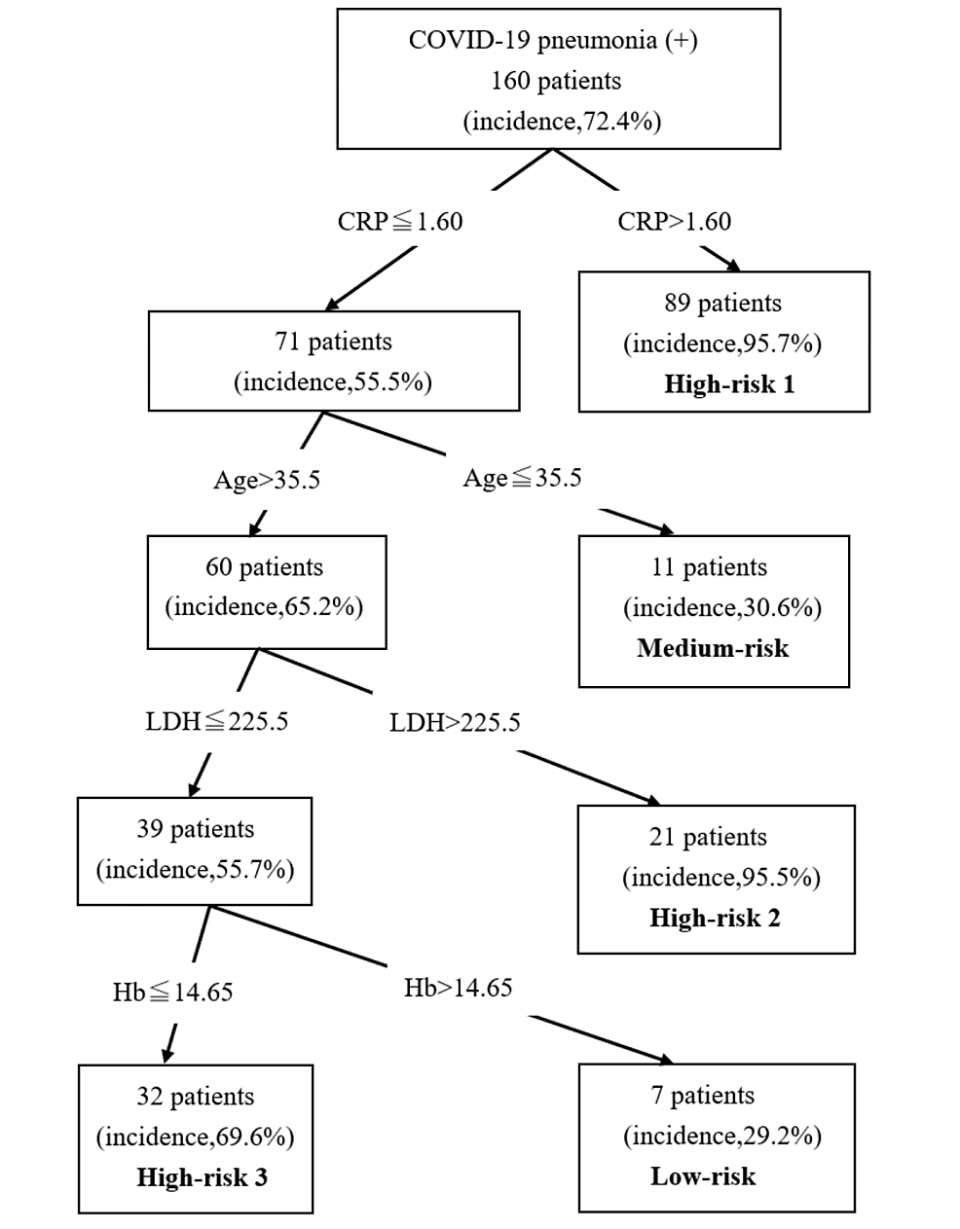
Decision tree for COVID-19 patients complicating pneumonia using classification and regression tree (CART) analysis. The results were derived from CART analysis. Low-risk (≤30%), medium-risk (30-60%), and high-risk (>60%). COVID-19, coronavirus disease 2019; CRP, C-reactive protein; LDH, lactate dehydrogenase; Hb, hemoglobin.

**Figure 2:**
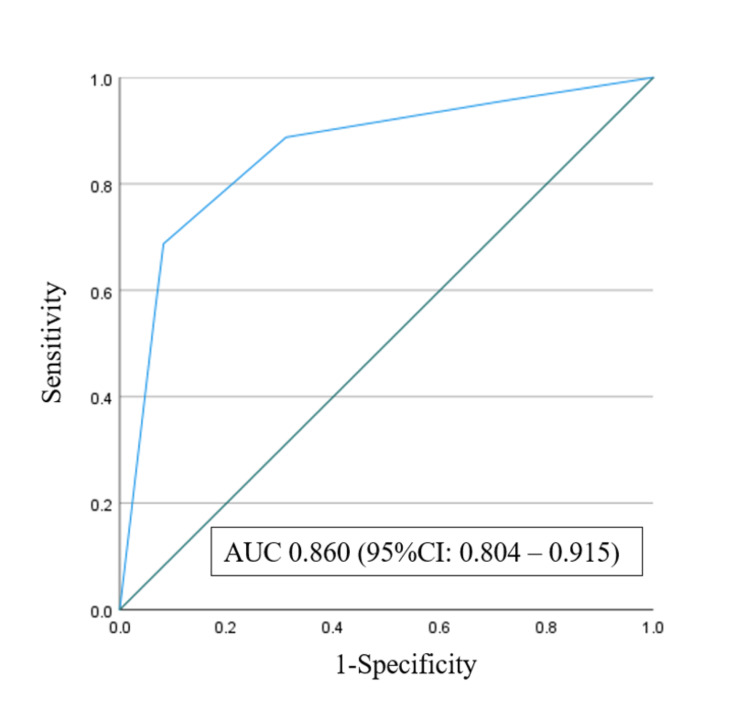
The area under the receiver operating characteristic curve of the model was 0.860 (95% confidence interval, 0.804–0.915). AUC, area under the curve

The algorithm for predicting pneumonia derived from CART is shown in Figure 1. The CRP level (≤1.60 mg/dL, >1.60 mg/dL), age (≤35.5 years, >35.5 years), LDH (≤225.5 IU/L, >225.5 IU/L), and hemoglobin (Hb) (≤14.65 g/dL, >14.65 g/dL) were included in the decision tree, and five terminal nodes were employed. Based on preliminary research using the CART analysis, we categorized the patients into three risk groups: low risk (≤30%), medium risk (30%-60%), and high risk (>60%) [12]. According to the risk groups, we divided these nodes into the following five categories: low risk (29.2% incidence of COVID-19 pneumonia), medium risk (30.6% incidence of COVID-19 pneumonia), high risk 1 (95.7% incidence of COVID-19 pneumonia), high risk 2 (95.5% incidence of COVID-19 pneumonia), and high risk 3 (69.6% incidence of COVID-19 pneumonia) (Figure 1). We evaluated the quality of this model using the ROC curve, which yielded an AUC of 0.860 and a 95% CI of 0.804-0.915 (Figure 2).

## Discussion

We directly compared COVID-19 patients with and without pneumonia using CART analysis to determine which patients should undergo CT. The four identified predictors were CRP, age, LDH, and Hb levels. High-risk patients should undergo CT. The area under the ROC curve demonstrated acceptable accuracy.

CRP is a pentameric protein synthesized by the liver whose levels increase with inflammation. It is an acute-phase protein that responds to inflammatory cytokines associated with monocytes or macrophages activated after infection. CRP is primarily induced by the action of interleukin-6, which is responsible for CRP gene transcription. In some cases, it activates the complement system, forming inflammatory cytokines, thereby further aggravating tissue damage [16]. The CRP level correlates with CT findings and can predict severe COVID-19 because of its significant increase at the initial stage of the disease [17]. The CRP level is also positively correlated with the diameter of lung lesions and severe presentation [18]. We believe that these findings are consistent with our results. Furthermore, our study included cut-off values (e.g., CRP level ≤1.60 mg/dL), which are useful in determining whether CT should be performed. Furthermore, obtaining daily CRP values for hospitalized COVID-19 patients can provide early thresholds and facilitate risk stratification and prognostication [19]. Therefore, it is important to monitor changes in CRP levels in COVID-19 patients.

LDH is an intracellular enzyme found in the cells of almost all organ systems that catalyzes the inter-conversion of pyruvate and lactate, with concomitant inter-conversion of NADH and NAD+ [20]. The enzyme is composed of subunits A and B and is present in humans as five separate isozymes (LDH-1, LDH-2, LDH-3, LDH-4, and LDH-5 in the cardiomyocytes, reticuloendothelial system, pneumocytes, kidneys and pancreas, and liver and striated muscles, respectively). Although LDH has been traditionally used since the 1960s as a marker of cardiac damage, abnormal values can result from multiple organ injuries and decreased oxygenation, along with upregulation of the glycolytic pathway. The acidic extra-cellular pH resulting from increased lactate from infection and tissue injury triggers the activation of metalloproteases and enhances macrophage-mediated angiogenesis [21]. Furthermore, severe infections may cause cytokine-mediated tissue damage and LDH release. The LDH type present in lung tissues is LDH-3. Patients with severe COVID-19 infections can release greater amounts of LDH in the circulation, often manifesting as acute respiratory distress syndrome, which is the hallmark of the disease [22]. Moreover, compared to non-COVID-19 pneumonia patients, COVID-19 pneumonia patients have higher AST, ALT, LDH, γ-glutamyl transpeptidase (γ-GT), and α-hydroxybutyric dehydrogenase levels [23]. Although AST, ALT, and γ-GT levels were not predictors in this CART analysis, we identified an LDH level of >225.5 IU/L as a predictive factor and one of the high-risk factors for COVID-19 pneumonia (high risk 2). Furthermore, in other studies, elevated LDH levels were associated with severe disease and mortality in COVID-19 patients [22]. Elevated LDH levels seem to indicate that multiple organ injury and failure may play a more prominent pathological role in influencing clinical outcomes in COVID-19 patients [22]. Therefore, LDH levels should be closely monitored, as they affect the overall status and course of the disease.

LDH and CRP levels may be related to respiratory function (PaO_2_/FiO_2_) and may predict respiratory failure in COVID-19 patients. LDH and CRP should be considered useful for identifying patients who require closer respiratory monitoring and more aggressive early supportive therapies to avoid poor prognoses [24]. LDH and CRP are important in predicting not only the presence but also the severity of pneumonia.

The mechanisms of Hb reduction during sepsis vary and may include altered microcirculation, decreased red blood cell (RBC) production, pre-existing chronic anemia, hemodilution, and increased RBC destruction due to altered RBC membranes [24]. In addition, the relationship between low initial Hb levels and mortality in conditions such as septic shock has been highlighted in other studies, and early treatment of patients with low initial Hb levels is thought to contribute to a reduction in mortality. However, the cut-off value of the Hb level in this study was within the normal range (14.65 g/dL). This topic should be further discussed in future studies. Similarly, the results for age in this study will be controversial in future. Other studies have reported that the age at the onset of COVID-19 is lower than that of influenza [25]. We believe that this reflects the strong infectivity of COVID-19, regardless of medical history and strength of immunity. This topic should also be discussed in future studies.

Similar studies on predictors of COVID-19 severity have also been performed in other countries [26], in which CRP, LDH, neutrophil-to-lymphocyte ratio, age, lymphocyte count, and malignancy were found to be associated with intubation using chi-square automatic interaction detection analysis [26,27]. It is interesting that CRP and the LDH were also predictors of disease severity in our study.

This study only included COVID-19 cases before any vaccine became widely available. Although they are not the correct data all over Japan in 2021, the overall prevalence of vaccine hesitancy was 5.5% in working-age adults in Japan [28]. As vaccines become even more ubiquitous, the efficacy of pneumonia as a predictor of COVID-19 will most likely change in future (however, it should be mentioned that the present study is not a current omicron stock).

Furthermore, COVID-19 was phenotypically milder in Japan than in other countries despite its application of relatively less restrictive preventive measures. Factors related to a possible reduced susceptibility to the pulmonary manifestations of SARS-CoV-2 may have contributed to better outcomes and lower mortality in Japan [29]. In addition, the treatment during this research period was not yet established in the world, including Japan. However, there are now established treatments, including Remdesivir and others [30]. Thus, it may be possible that the results may be at odds with the results as of 2023. The strength of our study is sensitizing all physicians to consider COVID-19 pneumonia, which remains an ongoing challenge. Early detection of pneumonia should improve management and decrease mortality in COVID-19 patients. There has been no reported analysis of factors predictive of COVID-19 pneumonia using CART analysis. We aimed to develop precise criteria for performing CT tests in COVID-19 patients. Our study also had some limitations. The study population was limited to a single hospital. Additionally, this was a retrospective study, and COVID-19 variants were not discussed. Therefore, we propose conducting a multicenter prospective study with a larger number of patients in the future. Additionally, we did not calculate the sample size because all COVID-19 patients who underwent blood tests and CT at our hospital between January 1, 2020, and December 31, 2020, were included. Prospective studies in the future must calculate the sample size beforehand.

Furthermore, due to the nature of CART, it is considered a limitation that the items and cut-off values are determined by purity, so it is impossible to evaluate the impact of each complication and how much it affects the patients. There is a mix of severely ill and mildly ill patients who will be admitted to the ICU in this study, and if similar studies were conducted only on severely ill patients and mildly ill patients, respectively, the results may differ.

This study did not use a questionnaire. Our model should also be validated in the future.

## Conclusions

We aimed to compare clinical parameters in patients with and without complicated pneumonia to identify predictive factors for COVID-19 patients with complicated pneumonia and determine which COVID-19 patients should undergo CT. 

We propose a predictive model based on CART analysis for pneumonia in patients with COVID-19, consisting of four predictors: CRP level, age, LDH level, and Hb level. The CART model is well suited to the generation of clinical decision rules and has been used to develop prediction models in various fields, including in medical settings. We emphasize that our study is a new predictive model using CART analysis and our results are extremely useful in deciding whether to perform CT in COVID-19 patients. 
